# Efficacy, Tolerability, and Safety of Fluoxetine for Panic Disorder in Adults: A Review

**DOI:** 10.7759/cureus.116516

**Published:** 2026-09-18

**Authors:** Haseeb Mohammad, Sehasbir Singh, Inderbir Singh

**Affiliations:** 1 Counselling Psychology, Yorkville University, Fredericton, CAN; 2 Counselling Psychology, University of Alberta, Edmonton, CAN; 3 Pharmacology, Chitkara University, Patiala, IND

**Keywords:** adverse effects, cyp2d6, drug delivery, fluoxetine, panic disorder, personalized medicine, pharmacogenetics, selective serotonin reuptake inhibitors, tolerability, treatment optimization

## Abstract

Fluoxetine is a selective serotonin reuptake inhibitor (SSRI) used in the pharmacological treatment of panic disorder. Its efficacy is supported by clinical trials, but the response to treatment and tolerability vary between patients, driving interest in strategies that could improve its clinical use. This review evaluates the effectiveness and side effects of fluoxetine in panic disorder, discusses clinical, pharmacokinetic, and pharmaceutical barriers to treatment, and assesses current and emerging strategies for treatment optimization.

We conducted a structured literature review drawing on two complementary streams of evidence. We reviewed the clinical literature for evidence of fluoxetine efficacy, response, remission, maintenance, adverse effects, tolerability, and discontinuation in panic disorder. A second stream was dedicated to fluoxetine-specific research on pharmacokinetics, pharmacogenetics, formulations, and drug delivery. Clinical conclusions were based on randomized controlled trials and systematic reviews, whereas preclinical and physicochemical investigations were separately addressed as potential future approaches.

Randomized trials support the efficacy of fluoxetine for panic disorder across panic symptoms, anxiety, phobic symptoms, global improvement, and functioning. Placebo-controlled evidence suggests that fluoxetine may increase the likelihood of achieving panic-free status during treatment. Fluoxetine has generally been well tolerated in controlled studies, although early activation, gastrointestinal effects, insomnia, and treatment intolerance were noted in some patients. Cytochrome P450 2D6 (CYP2D6) contributes to variability in fluoxetine and norfluoxetine concentrations, but there is insufficient evidence to support the routine use of genotype to guide dosing. Cocrystals, nanoparticles, and intranasal delivery systems can alter physicochemical properties or drug distribution but have not been demonstrated to be clinically helpful in panic disorder.

Fluoxetine is a pharmacological option with evidence of efficacy in panic disorder. The clinical value of fluoxetine depends on a balance between efficacy and tolerability. Optimization now should focus on individualized initiation and titration of doses, management of adverse effects, adherence, drug interactions, patient preferences, and ongoing monitoring. Pharmacogenetics, therapeutic drug monitoring, and advanced drug delivery systems are still promising areas of investigation for the future rather than established clinical strategies.

## Introduction and background

Evaluating treatment outcomes in panic disorder

Psychiatric medications should not be evaluated on the basis of whether they achieve statistically significant reductions in symptoms. Additionally, treatment response should be evaluated in terms of remission, functional improvement, adverse effects, treatment adherence, and patients’ ability to continue treatment [1,2]. This is particularly important in the case of panic disorder, where the physical sensations associated with medication may themselves become distressing to those already sensitized to bodily symptoms associated with anxiety and panic. Thus, both efficacy and tolerability of a pharmacological treatment need to be assessed to evaluate its overall clinical value [1].

Panic disorder

Panic disorder is defined as recurrent, unexpected panic attacks, which are abrupt surges of intense fear or discomfort that may include cardiovascular, respiratory, gastrointestinal, neurological, and cognitive symptoms [1,3]. People may also fear future panic attacks and change their behaviour to avoid situations that they believe may trigger the attacks. These symptoms may interfere with social, occupational, and other important areas of functioning [3]. Psychological interventions, especially cognitive behavioural therapy (CBT), and pharmacological interventions are commonly used in treatment. Antidepressants, especially selective serotonin reuptake inhibitors (SSRIs), have emerged as the first-line pharmacological treatment because of their effectiveness and improved tolerability compared to older antidepressants such as tricyclic antidepressants and monoamine oxidase inhibitors [1,3,4].

There is much evidence for antidepressant treatment, but this does not necessarily mean that all patients respond in the same way. Bighelli et al. [1], in a Cochrane review of 41 randomized controlled trials with 9,377 participants, found that antidepressants reduced the risk of treatment nonresponse compared with placebo. The number needed to treat one extra patient to benefit was around seven. However, as antidepressants are associated with a higher rate of discontinuations due to adverse effects, efficacy should be balanced with tolerability. In addition, incomplete response, relapse of symptoms, adverse effects, and discontinuation of treatment continue to be important challenges in the pharmacological management of panic disorder [5,6].

Fluoxetine: uses and mechanism of action

Fluoxetine is an SSRI and potently inhibits the serotonin transporter, resulting in decreased presynaptic serotonin reuptake and, consequently, increased synaptic cleft availability of serotonin. Fluoxetine is also metabolized into the pharmacologically active norfluoxetine, and the prolonged elimination of both compounds contributes to the relatively long pharmacokinetic profile [7]. In panic disorder, fluoxetine has been studied in low starting doses with gradual increases [8]. This is especially relevant as some patients with panic disorder may be sensitive to early activation or other adverse effects [9,10].

Panic attacks are a hallmark feature of panic disorder, but panic attack frequency is not the only measure of treatment success [2]. Fluoxetine studies have measured outcomes of panic-free status, global anxiety, anticipatory anxiety, phobic avoidance, global clinical improvement, depressive symptoms, and functional impairment [2,8]. Thus, several clinical outcomes were investigated to more fully understand its therapeutic effects [2].

In addition to clinical efficacy and adverse effects, treatment outcomes may be affected by pharmacokinetic variability, drug interactions, adherence, formulation characteristics, and patient preferences [3,11,12]. Recent research on cytochrome P450 2D6 (CYP2D6) variability, pharmacogenetics, therapeutic drug monitoring, cocrystals, nanoparticles, and intranasal delivery has opened up potential avenues for further optimization of treatment [11-16]. But these strategies are fundamentally different in terms of evidence. Pharmacokinetic variability has been shown in humans, although many sophisticated formulation and drug delivery approaches are still confined to physicochemical or preclinical studies [11-16].

Purpose and clinical importance of the review

This review discusses the efficacy and adverse effects of fluoxetine in panic disorder, describes clinical, pharmacokinetic, and pharmaceutical challenges that may affect its clinical use, and highlights emerging strategies for treatment optimization. Individualized dose titration and adverse effect management are highlighted as well-established clinical practices, contrasted with new approaches like pharmacogenetics, pharmaceutical formulation, nanotechnology, and targeted drug delivery.

## Review

Methods

Study Design

This study used a structured literature review method to explore evidence on efficacy, adverse effects, and treatment optimization of fluoxetine in panic disorder. The review addresses clinical psychopharmacology and optimization of pharmaceutical treatment; therefore, the literature was considered under two complementary streams: clinical evidence for the treatment of panic disorder with fluoxetine and pharmaceutical evidence for the pharmacokinetics, pharmacogenetics, formulation, and drug delivery of fluoxetine.

Data Collection

The search strategy involved using academic databases (i.e., PubMed, PsycINFO, Embase, Cochrane Library, and Scopus) to identify relevant literature on fluoxetine and panic disorder. In addition, the reference lists of relevant clinical trials, systematic reviews, meta-analyses, and clinical guidelines were reviewed for potentially relevant studies. Clinical search terms included “fluoxetine”, “Prozac”, “panic disorder”, “panic attacks”, “agoraphobia”, “efficacy”, “response”, “remission”, “relapse”, “maintenance”, “adverse effects”, “tolerability”, “safety”, and “discontinuation”. For treatment optimization literature, a different search strategy was employed using combinations of “fluoxetine”, “norfluoxetine”, “pharmacokinetics”, “CYP2D6”, “pharmacogenetics”, “therapeutic drug monitoring”, “formulation”, “solubility”, “bioavailability”, “blood-brain barrier”, “intranasal”, “nose-to-brain”, “nanoparticles”, “nanocarriers”, “cubosomes”, “cocrystals”, and “drug delivery”. The two search streams were treated separately, as formulation and drug delivery research may not include participants with panic disorder but may contain fluoxetine-specific information relevant to future pharmaceutical optimization.

Following peer review, additional literature on virtual reality exposure therapy (VRET) was considered to provide contextual evidence regarding emerging non-pharmacological interventions for panic and anxiety disorders. These studies were considered separately from the fluoxetine-specific evidence and were not used to draw conclusions regarding the efficacy or tolerability of fluoxetine.

Inclusion and Exclusion Criteria

The inclusion criteria were clinical trials in adults with panic disorder with or without agoraphobia that evaluated fluoxetine. Randomized controlled trials were preferred in the assessment of efficacy and adverse events. We also included active comparator studies, continuation studies, systematic reviews, meta-analyses, and network meta-analyses that provided relevant efficacy, tolerability, comparative treatment performance, maintenance, or relapse information. Open-label studies were included if they provided clinically relevant information regarding dosing, activation, early tolerability, or continuation of treatment not adequately represented in controlled studies.

Studies examining SSRIs or antidepressants as a whole were included for clinical context but kept separate from evidence specific to fluoxetine. For instance, Bighelli et al. [1] included randomized placebo-controlled trials across several classes of antidepressants and analyzed SSRIs separately, with only limited fluoxetine-specific data for some individual outcomes.

Human pharmacokinetic and pharmacogenetic studies were included if they evaluated fluoxetine or norfluoxetine concentrations, cytochrome P450 (CYP) genotypes or phenotypes, therapeutic drug monitoring, or other sources of pharmacokinetic variability. Since fluoxetine metabolism is drug-specific and not diagnosis-specific, these studies did not have to involve panic disorder. However, clinical findings from other diagnostic populations were not assumed to directly establish treatment outcomes in panic disorder.

Only formulation studies specifically for fluoxetine using cocrystals, nanoparticles, intranasal systems, or other drug delivery methods were included. Preclinical and in vitro studies were separated from clinical research and not used as evidence of a formulation improving efficacy or tolerability in patients with panic disorder.

We excluded studies that did not provide information relevant to fluoxetine or the treatment of panic disorder, secondary publications that simply summarized an available primary study, or when the same study was duplicated in the collected literature. Studies focusing on other disorders only were excluded from the clinical efficacy analysis.

Data Analysis

A narrative synthesis was performed due to the marked heterogeneity of the included literature regarding study design, treatment duration, dose, comparator, population, and outcome measures. Clinical evidence was grouped according to acute efficacy, maintenance and relapse, adverse effects and tolerability, and global benefit-risk balance. Randomized placebo-controlled trials, systematic reviews, and meta-analyses were weighted more in judging clinical efficacy.

Pharmacokinetic and pharmacogenetic findings were analyzed separately to explore whether patient-to-patient differences have the potential to provide opportunities for individualized treatment. Formulation and drug delivery studies were evaluated based on the level of evidence, and physicochemical and animal findings were interpreted as potential approaches for future optimization of treatment rather than established clinical benefits.

Results and discussion

Efficacy of Fluoxetine in Panic Disorder

Early studies have indicated that fluoxetine may reduce panic symptoms but also point out the limitations of uncontrolled evidence [9,10]. Gorman et al. [9] conducted an open trial with patients with panic disorder or agoraphobia with panic attacks. Among the study participants, seven responders showed complete cessation of panic attacks. However, eight of the nine nonresponders were not able to tolerate the adverse effects [9]. These results provide some preliminary evidence for an antipanic effect and at the same time illustrate the importance of tolerability and controlled research [9,10].

Similarly, Pecknold et al. [10] reported significant reductions in panic attacks, anxiety, and phobic avoidance during eight weeks of fluoxetine treatment. By week eight, about 48% of those who began treatment were free of panic attacks. But ~36% discontinued because of worsening anxiety or lack of efficacy. This initial research suggests possible therapeutic value but also problems of activation, dose acceptance, and no placebo controls [10].

Later, randomized controlled trials provided stronger evidence. Michelson et al. [2] randomly assigned 243 adults with panic disorder to receive fluoxetine 10 mg/day, fluoxetine 20 mg/day, or placebo. Fluoxetine (especially at 20 mg/day) resulted in improvements in several outcomes, including global clinical improvement, panic attacks, anxiety, phobic symptoms, depressive symptoms, and functional impairment. However, at the final acute-treatment assessment, complete panic-free status did not differ significantly across all treatment groups. This finding is important as it indicates that treatment benefit may be observed across broader dimensions of panic disorder even when a single binary outcome does not reach statistical significance.

Michelson and colleagues [8] also examined the clinical effectiveness of fluoxetine using a dosing regimen of 10 mg/day for the first week and then increasing to 20 mg/day. Patients with suboptimal response after six weeks could be escalated to a further dose. Fluoxetine was superior to placebo for improving several outcomes. At week six, 29% of patients receiving fluoxetine were free of panic versus 16% on placebo; by study endpoint, the proportions were approximately 42% and 28%, respectively. Additionally, about 82% of patients receiving fluoxetine had a reduction of 50% or more in panic attacks compared with 61% of placebo patients.

This finding was subsequently supported by Bighelli et al. [1]. In their Cochrane review, Bighelli et al. [1] included Michelson et al. [8] and reported a fluoxetine versus placebo risk ratio (RR) of 0.46 (95% CI 0.27-0.76) for failure to respond, suggesting fluoxetine was associated with a smaller proportion of patients failing to respond than placebo [1]. This estimate for fluoxetine, however, came from a single study in 180 participants and is not evidence from multiple independent trials of fluoxetine [1].

Bighelli et al. [1] also found an RR of 0.80 (95% CI 0.64 to 1.00) for failure to remit for fluoxetine compared with placebo. Continuous outcomes in the same trial showed significant improvement in panic symptoms (standardized mean difference -0.61, 95% CI -0.91 to -0.31). The review also noted that the Michelson et al. [8] study showed significant improvement in broader outcomes of anxiety and social functioning. These findings add to the argument that the effect of fluoxetine should be assessed across panic symptoms, anxiety, avoidance, and functioning, and not only on panic frequency.

Supportive evidence also exists at the broader class of antidepressants level. Antidepressants reduced treatment nonresponse compared with placebo, with an overall RR of 0.72 and an estimated number needed to treat of seven [1]. For SSRIs specifically, the risk of nonresponse was reduced compared with placebo (RR 0.75, 95% CI 0.67-0.84), and SSRIs also reduced failure to remit (RR 0.81, 95% CI 0.75-0.88) [1]. Class-level findings are consistent with the pharmacological use of fluoxetine but should not be interpreted as fluoxetine-specific findings.

Similarly, Andrisano et al. [17] found that newer antidepressants were effective in panic disorder, and fluoxetine resulted in significantly greater improvement than placebo for panic and overall anxiety outcomes. However, the differences in methodology between the included studies, plus the inclusion of some uncontrolled evidence, limit the degree of precision with which individual drugs can be ranked.

More recent network meta-analyses also support treatment with antidepressants, with little evidence to suggest fluoxetine is uniquely superior. Fluoxetine was found to be efficacious compared with placebo, but other pharmacological treatments were also found to have favorable efficacy and acceptability [18]. Chawla et al. [19] found that SSRIs as a class improved remission compared with placebo, and sertraline and escitalopram appeared particularly favorable when considering efficacy and adverse events together. The pharmacological treatment of panic disorder was supported in the 2023 Cochrane network meta-analysis of Guaiana et al. [20], who also assessed fluoxetine among the interventions. But the accuracy of the estimates for individual drugs was inconsistent, underscoring that rankings of treatments should not be considered as evidence of superiority.

To summarize, the evidence suggests that fluoxetine is clinically effective in panic disorder, especially when treatment response is assessed by panic attacks, global anxiety, phobic avoidance, and functioning. The available evidence, however, does not suggest fluoxetine consistently performs better than other SSRIs.

Comparative Efficacy

Head-to-head studies give us more information about where fluoxetine fits when compared with other treatments [21]. Fluoxetine was compared with moclobemide in 366 patients by Tiller et al. [21]. At week eight, roughly 70% of the fluoxetine-treated subjects and 63% of those treated with moclobemide were panic-free. This difference was not statistically significant. Patients who continued therapy generally maintained clinical benefit; however, long-term completion data should be interpreted with caution as patients who remain on extension studies may differ from those who drop out [21].

In a small randomized double-blind trial, Ribeiro et al. [22] compared fluoxetine with mirtazapine. Both groups improved on most clinical outcomes, and there were no significant between-group differences for panic attack frequency, Hamilton Anxiety scores, or Sheehan Phobic Scale [23,24]. One measure, patient global evaluation of phobic anxiety, favored mirtazapine. The drugs also differed in adverse effect profiles, meaning similar efficacy does not necessarily mean similar experience for patients [22]. The comparative data thus further support the overall conclusion that fluoxetine is an effective pharmacological option but do not show a clear advantage over available alternatives.

The key clinical studies supporting the efficacy and tolerability findings discussed above are summarized in Table 1.

**Table 1:** Summary of key evidence on fluoxetine in panic disorder. RCTs: randomized controlled trials; NNTB: number needed to treat for an additional beneficial outcome; SSRI: selective serotonin reuptake inhibitor; RR: risk ratio.

Study	Design	Population	Main findings	Key limitation
Gorman et al. [9]	Open trial	20 patients were enrolled; four placebo responders were excluded before fluoxetine treatment, leaving 16 patients in the fluoxetine phase	Seven responders had complete cessation of panic attacks; eight of nine nonresponders could not tolerate adverse effects	Small, uncontrolled study
Pecknold et al. [10]	Open-label eight-week study	30 adults with panic disorder were enrolled; 28 began fluoxetine treatment	Reduced panic attacks, anxiety, and phobic avoidance; 48% panic-free by week eight; ~36% discontinued	No placebo control
Michelson et al. [2]	Randomized placebo-controlled trial	243 adults with panic disorder	Fluoxetine, especially 20 mg/day, improved global improvement, panic, anxiety, phobic symptoms, depressive symptoms, and functioning; panic-free status not significantly different at endpoint	Final panic-free outcome was not statistically significant
Michelson et al. [8]	Randomized placebo-controlled trial	180 adults with panic disorder	29% vs 16% panic-free at week six; ~42% vs 28% at endpoint; 82% vs 61% achieved ≥50% panic reduction	Short-term trial
Tiller et al. [21]	Head-to-head trial	366 patients	Fluoxetine was similar to moclobemide; ~70% vs 63% panic-free at week eight	No placebo arm
Ribeiro et al. [22]	Small randomized double-blind trial	27 patients	Fluoxetine was similar to mirtazapine on most outcomes; adverse effect profiles differed	Very small sample
Bighelli et al. [1]	Cochrane review	41 RCTs, 9,377 participants	Antidepressants: NNTB approximately seven; SSRI class improved response/remission; fluoxetine-specific RR for failure to respond 0.46 based on one study	Mostly class-level, not fluoxetine-specific

Maintenance Treatment and Relapse

Panic disorder can be recurrent, and maintenance of treatment response is of clinical importance. Freire and Nardi [5] identified relapse prevention as an important component in the long-term management of panic disorder. The long-term evidence for individual medications, however, is much smaller than the acute treatment literature.

Tiller et al. [21] included a longer-term extension in which patients continuing treatment generally maintained significant clinical improvement. However, extension phase analyses are largely conducted in patients who have responded to and tolerated therapy and are therefore less definitive than randomized maintenance studies.

Weekly fluoxetine was studied in 10 patients who were panic-free while receiving fluoxetine daily [25]. All 10 were successfully converted to weekly dosing, with follow-up up to 26 months and one subsequent recurrence. The small, uncontrolled nature of the sample precludes firm conclusions from being drawn. However, the study reflects how the long pharmacokinetic profile of fluoxetine has generated interest in alternative dosing strategies to improve convenience or adherence [25].

​​These findings suggest that treatment should not be solely focused on an acute response for optimization. Maintenance, adherence, recurrence, and the feasibility of continuation of treatment are other important aspects of successful pharmacotherapy.

Adverse Effects, Safety, and Tolerability

The adverse effect profile of fluoxetine is somewhat different in early uncontrolled studies from later randomized trials [2,8-10]. Initial studies indicated clinically significant activation and treatment intolerance in a subset of patients [9,10]. Gorman et al. [9] found that eight of nine nonresponders were unable to tolerate adverse effects, and Pecknold et al. [10] reported discontinuation due to increased anxiety or lack of efficacy. These findings raised the concern that patients with panic disorder may be particularly sensitive to early medication-related activation [9,10].

Randomized trials later demonstrated, however, a better tolerability profile when fluoxetine was initiated and titrated more slowly. Michelson and colleagues [8] found that relatively few people stopped the medication because of side effects. The side effects that people reported were nausea, tremor, headache, vomiting, and insomnia. Similarly, Michelson et al. [2] found no significant difference in discontinuation due to adverse events between fluoxetine and placebo.

Bighelli et al. [1] made a useful distinction between tolerability of the SSRI class and fluoxetine-specific tolerability. Bighelli et al. [1] found that SSRIs were associated with more discontinuations due to adverse effects than placebo (RR 1.45, 95% CI 1.16-1.81). But the fluoxetine-specific estimate was much less certain. Discontinuation of Michelson et al. [8] due to adverse effects produced an RR of 1.67 (95% CI 0.41-6.77), and experiencing at least one adverse effect produced an RR of 1.32 (95% CI 0.78-2.21). No difference was found in either confidence interval. The more general SSRI finding should not be used as evidence that fluoxetine itself definitely causes a 45% increase in adverse effect discontinuation [1].

Adverse effects were relatively common with both fluoxetine and moclobemide [21] and included nausea, headache, dizziness, diarrhea, fatigue, and dry mouth. Ribeiro et al. [22] further illustrated the significance of the type of adverse effect as well as the occurrence of adverse effects. Fluoxetine resulted in more nausea and paresthesia, and mirtazapine resulted in more weight gain.

Another methodological issue, raised by Bighelli et al. [26], was whether participants experiencing adverse effects may be able to correctly guess that they are receiving active medication, thus inflating apparent antidepressant efficacy via expectancy effects. Their analysis showed that antidepressant treatment was associated with response even after adjusting for adverse events, offering little support for the concept that the efficacy found in panic disorder antidepressant trials is attributable solely to unblinding caused by adverse effects.

In general, fluoxetine seems to be reasonably well tolerated by many patients, especially if introduced slowly [8,10]. However, early activation, gastrointestinal effects, sleep disturbance, and other adverse effects can have a significant impact on individual treatment experience and adherence [9,10,21].

Discontinuation and Withdrawal

Adverse events during active treatment should be differentiated from symptoms related to dose reduction or discontinuation [27]. Henssler et al. [27] found withdrawal symptoms to be a significant problem across antidepressants but a relatively lower risk with fluoxetine versus several antidepressants with a shorter half-life.

The long half-lives of elimination of fluoxetine and its metabolite norfluoxetine lead to a slow decline in concentrations after stopping the drug [7,27]. This pharmacokinetic property may reduce the risk of sudden discontinuation symptoms but does not mean that withdrawal or recurrence are impossible [5,27]. Therefore, when evaluating symptoms after treatment discontinuation, withdrawal symptoms, rebound symptoms, and recurrence of panic disorder should be considered separately [5,27].

Balancing Efficacy and Adverse Effects

The clinically relevant question is not simply whether fluoxetine is associated with adverse effects but whether its expected therapeutic benefits outweigh those harms relative to placebo and available alternative treatments [1,8,21,22]. This balance at the antidepressant-class level is clearly illustrated by Bighelli et al. [1]. Antidepressants reduced failure to respond (estimated number needed to treat for an additional beneficial outcome (NNTB) of seven) but also increased discontinuation because of side effects. SSRIs had lower nonresponse and nonremission rates and no difference in dropout rates compared with placebo, but there was a higher incidence of discontinuation due to adverse effects [1].

This pattern supports an assessment of treatment on the basis of a benefit-risk profile rather than merely the characterization of a medication as effective or ineffective [1]. One patient may have a very good response to fluoxetine, with substantial reduction in symptoms and acceptable side effects; another may discontinue due to early activation or persistent side effects before a therapeutic response can be achieved [8-10]. Therefore, treatment optimization should take into account efficacy and tolerability at the same time [1].

Pharmacokinetic Variability as a Treatment Challenge

Interindividual pharmacokinetic variability is also a challenge for fluoxetine [11,13]. Fluoxetine is metabolized through multiple CYP pathways into the active metabolite norfluoxetine, with CYP2D6 being important [28]. The genetic variation in CYP2D6 activity produces different metabolizer phenotypes, which can change the relative concentrations of fluoxetine and norfluoxetine [11,28].

Nordby et al. [11] examined CYP2D6 genotype and fluoxetine exposure in more than 1,000 adults. The CYP2D6 phenotypes showed different relationships between fluoxetine and norfluoxetine concentrations. However, the combined active levels of fluoxetine and norfluoxetine were not significantly different between the metabolizer groups [11]. This finding is clinically relevant because differences in the individual parent and metabolite concentrations are not necessarily indicative of major differences in total pharmacologically active exposure [11,28].

Nevertheless, Nordby et al. [11] found that the odds for adults who were ultrarapid and poor metabolizers to switch from fluoxetine to another antidepressant were approximately two to three times higher than those of normal metabolizers. The reason for switching was unknown, and the findings cannot determine whether switching was due to lack of efficacy, adverse effects, clinician preference, or other reasons [11].

de la Cruz et al. [13] provided further evidence from real-world practice on the impact of CYP2D6. CYP2D6 genotype was a significant predictor of the dose-normalized fluoxetine to norfluoxetine metabolic ratio, but cytochrome P450 2C9 (CYP2C9), cytochrome P450 2C19 (CYP2C19), and the clinical variables studied were not significant independent predictors [13].

Despite this pharmacokinetic evidence, the clinical utility of routine pharmacogenetic testing for fluoxetine is still unclear [28]. The Clinical Pharmacogenetics Implementation Consortium (CPIC) guideline by Bousman et al. [28] acknowledges the influence of CYP2D6 on fluoxetine metabolism but does not currently offer a CYP2D6 genotype-based fluoxetine dosing recommendation. This is due in part to the fact that both fluoxetine and norfluoxetine are active, multiple metabolic pathways contribute to disposition, and genotype has not shown a consistent enough relationship to treatment efficacy or adverse effects [28].

Therefore, CYP2D6 is a reproducible source of pharmacokinetic variability, and there is no current evidence to support routine CYP2D6-guided fluoxetine dosing [11,13,28]. Pharmacogenetic information is better conceived as a promising area of further treatment optimization than as an established clinical solution [13,28].

Dosage Forms and Treatment Flexibility

Fluoxetine is available in conventional oral formulations, and formulation flexibility may influence adherence and patient preference [29]. The standard approach is daily capsules or tablets, but where available, liquid formulations can offer greater flexibility in the measurement of the dose [29]. Theoretically, this could be useful for people who respond to smaller dose adjustments or have difficulty swallowing tablets or capsules.

The long pharmacokinetic profile of fluoxetine has also allowed for the possibility of studying less frequent dosing [25]. Emmanuel et al. [25] demonstrated that once-a-week dosing could be given to a very small group of patients with panic disorder who had already responded to daily fluoxetine. While the evidence is not sufficient to establish weekly dosing as an alternative standard, it illustrates how formulation and dosing convenience may affect patient adherence and receptivity [25].

Solubility and Cocrystal Approaches

Poor solubility cannot be regarded, on the basis of available evidence, as a major established clinical limitation of fluoxetine [12,14]. Rather, the more justifiable question is whether a change in its solid-state properties could alter dissolution and result in other pharmaceutical designs [12,14]. Fluoxetine hydrochloride has been reported to cocrystallize with a variety of organic acids [12]. The intrinsic dissolution properties of fluoxetine hydrochloride are modifiable by the selection of a coformer [12]. Importantly, some cocrystals enhanced dissolution while others decreased dissolution, indicating that cocrystallization is not a solution for pharmaceutical improvement [12]. Similarly, Parmar and Shah [14] studied fluoxetine hydrochloride cocrystals and found that a fluoxetine hydrochloride-maleic acid cocrystal presented enhanced solubility and dissolution under the experimental conditions used. These results demonstrate that crystal engineering can modulate the physicochemical properties of fluoxetine [12,14]. However, neither study showed improved bioavailability, panic-disorder efficacy, treatment tolerability, adherence, or adverse effects in patients with cocrystals [12,14]. Therefore, cocrystallization should be considered as a potential formulation approach, and not a solution to a clinical problem with conventional fluoxetine [12,14].

Blood-Brain Barrier and Drug Targeting

Care must also be taken in framing the blood-brain barrier permeability. Conventional oral fluoxetine clearly has central nervous system effects and has demonstrated clinical efficacy in psychiatric disorders including panic disorder [2,8]. Thus, it is not right to say that fluoxetine does not cross the blood-brain barrier adequately. Rather, the question of optimization is whether alternative delivery systems can alter the rate, magnitude, or localization of fluoxetine or the brain-to-systemic distribution [15].

Improved targeting to the brain could, in principle, enhance the delivery of a larger fraction of the administered drug to the relevant central nervous system tissues and minimize unnecessary systemic exposure [15]. This approach may ultimately provide opportunities to enhance efficacy, lower dose requirements, or reduce systemic adverse effects. However, these proposed benefits remain theoretical until demonstrated in the clinic [15].

Nano-Drug Delivery and Intranasal Strategies

Nanotechnology is one of the most experimental treatment optimization approaches discussed in this review and is a rapidly developing area of pharmaceutical research [15,16]. Jain et al. [15] formulated a thermoreversible fluoxetine-loaded cubosomal in situ gel for the intranasal route. The formulation showed good encapsulation efficiency, sustained release, increased nasal residence, and altered fluoxetine exposure in animal studies [15]. The authors reported enhanced bioavailability in comparison with cubosomal dispersion and drug solution [15]. The intranasal route is of particular interest, as it may allow the possibility of changing drug distribution through pathways involving the olfactory and trigeminal regions [15]. This enables the potential for increasing brain-directed exposure with the possibility of modifying systemic distribution [15]. However, Jain et al. [15] did not assess patients with panic disorder. The study was a pharmaceutical and preclinical study; thus, it cannot demonstrate that intranasal cubosomal fluoxetine improves antipanic efficacy, reduces adverse effects, or increases patient adherence [15].

Dadkhah et al. [16] investigated fluoxetine-loaded, polyethylene glycol-modified (PEGylated) chitosan nanoparticles in an animal model of hippocampal demyelination. The nanoparticle formulation modified the drug release and was associated with differences in behavioral and neurobiological outcomes compared with free fluoxetine [16]. While this provides evidence that nanoencapsulation can alter the delivery and pharmacological effects, the disease model is quite different from panic disorder and should not be used as evidence of improved panic treatment [16]. So current results in nanotech are evidence of pharmaceutical feasibility, not clinical superiority [15,16].

Drug Targeting and Reduction of Systemic Adverse Effects

In principle, drug targeting may improve the therapeutic index of centrally acting drugs by increasing drug exposure to the desired site of action while decreasing exposure to other tissues [15]. This suggests a possible research avenue for fluoxetine where targeted or intranasal delivery could reduce systemic drug exposure while maintaining or improving central nervous system exposure [15]. However, the literature does not currently provide any evidence that cocrystals, cubosomes, or nanoparticles reduce clinical fluoxetine toxicity or adverse effects in humans [12,14-16]. Therefore, the word toxicity should not be used as a synonym for common side effects like nausea, insomnia, or activation. In the clinical sections of this review, the more appropriate terms are adverse effects, safety, and tolerability. Toxicity should only be used for research that involves toxic exposure, overdose, organ injury, or formulation studies that show decreased toxic exposure.

Clinically Available Treatment Optimization

Emerging technologies are scientifically interesting, but the treatment optimization strategies that are most clinically applicable today are much simpler. One of the most obvious is dose initiation and titration [8,10]. Initial research by Gorman et al. [9] and Pecknold et al. [10] showed that some patients experience clinically significant activation or intolerance. Later studies with lower initial doses demonstrated more favorable tolerability [2,8]. For example, Michelson et al. [8] began fluoxetine treatment at 10 mg/day and then increased to 20 mg/day, with further increases allowed for patients who were still inadequately improved.

The available evidence therefore supports a treatment model based on: low initial dose → assessment of tolerability → evaluation of response → cautious titration when needed.

Additional factors to consider when personalizing therapy include prior response to therapy, concomitant conditions, concomitant medications, drug-drug interactions, barriers to adherence, risk of adverse effects, and patient preferences [3].

van Apeldoorn et al. [30] provide a useful broader treatment context. Their study was a comparison of CBT, SSRI treatment, and combined treatment in panic disorder. The results cannot be attributed specifically to fluoxetine because several SSRIs were allowed [30]. However, the study supports the importance of considering pharmacological treatment as part of a wider individualized treatment plan, rather than assuming that medication selection per se determines outcome [30]. National Institute for Health and Care Excellence (NICE) [3] also emphasizes the importance of shared decision-making and consideration of individual needs and preferences. In panic disorder, it may be particularly important to increase patient understanding of the expected time for benefit, possible early adverse effects, and the rationale for gradual dose escalation [3,8,10].

VRET

Non-pharmacological approaches may also complement pharmacotherapy in the treatment of panic and anxiety disorders. VRET provides controlled exposure to anxiety-provoking situations without medication exposure. A recent meta-analysis of 26 randomized controlled trials involving 1,649 participants found that VRET reduced anxiety and phobia symptoms and improved approach behavior in clinically diagnosed populations [31]. Zainal et al. [32] also reported reductions in social anxiety in a pilot randomized trial of self-guided VRET. More directly relevant to panic disorder, Jung et al. [33] evaluated personalized virtual reality exposure in patients with panic disorder and agoraphobia and demonstrated that individualized virtual reality scenarios could elicit stronger anxiety responses. However, this preliminary study did not establish sustained treatment efficacy. Therefore, VRET represents a promising non-pharmacological or adjunctive intervention, but further panic disorder-specific randomized trials are required before its role can be directly compared with fluoxetine or other pharmacological treatments.

Patient Receptivity

Whether an effective drug is taken for a sufficient length of time to achieve a therapeutic effect may depend on the willingness/receptivity of the patient. This is particularly relevant in panic disorder, where people may be very sensitive to bodily sensations similar to symptoms experienced during the course of a panic attack. For example, early medication effects such as temporary nausea, nervousness, sleep disturbance, or other such effects may be taken as signs that treatment is dangerous or that the disorder is getting worse. Therefore, anticipatory guidance may enable continued treatment. Ways to do this include explaining possible adverse effects before treatment, starting at a lower dose if clinically appropriate, slowly increasing the dose, scheduling early follow-up, involving the patient in decisions about dose adjustments, and choosing a dosage form that is practical for the individual. Personalization in this context does not require advanced technology. Patient-centered dosing and treatment communication are already clinically available forms of personalized care.

This challenge can also be understood through Bandura’s concept of self-efficacy, which refers to an individual’s perceived ability to carry out behaviors needed to achieve desired outcomes [34]. Self-efficacy may be particularly relevant in panic disorder because patients may need to tolerate feared bodily sensations while developing confidence in their ability to cope with panic-related experiences. In a study of 361 individuals receiving CBT for panic disorder, Gallagher et al. [35] found that changes in self-efficacy preceded and independently predicted subsequent changes in panic symptoms. These findings suggest that the development of coping confidence and mastery is an important consideration in the broader management of panic disorder.

Personalized Medicine

Fluoxetine is an interesting candidate for further individualized medicine research because treatment outcomes may be influenced by many patient-specific variables, including pharmacokinetics, genotype, susceptibility to adverse effects, adherence, comorbidities, concomitant medications, and patient preference. However, personalized healthcare should not be limited to just CYP2D6 testing. Pharmacogenetic evidence to date demonstrates biological variability, but no validated algorithm exists to define the best fluoxetine dose for each patient.

A more clinically realistic personalized framework would therefore incorporate: patient characteristics → initial dose → tolerability → clinical response → dose adjustment or treatment change → continued monitoring.

If shown to improve clinical outcomes, future pharmacogenetic and therapeutic drug monitoring approaches may ultimately be added to this process. Thus, fluoxetine should be considered as a promising candidate for further personalized treatment research, rather than as a pharmacogenetically guided treatment already established.

Challenges and Limitations of Current Evidence

There are some limitations in the interpretation of the available evidence. First, a lot of the direct fluoxetine-specific panic disorder evidence is quite old. Several important studies appeared in the late 1980s through the early 2000s. Early studies were often small and uncontrolled, and the larger randomized studies employed diagnostic frameworks and outcome measures that differ somewhat from those used in contemporary clinical research.

Second, systematic reviews show far more evidence for the classes of antidepressants and SSRIs than for fluoxetine as a single drug. Bighelli et al. [1] included 41 unique randomized trials with 9,377 participants, but their main fluoxetine-specific estimates were based on many fewer trials. The fluoxetine comparison for a number of outcomes was based solely on the Michelson et al. [8] study with 180 patients. Thus, it is not fair to show large sample sizes of the SSRI class when, in fact, thousands of participants were never exposed to fluoxetine in particular.

Third, study quality and funding should be considered when interpreting the available evidence. Bighelli et al. [1] rated much of the evidence as moderate or low quality because of inconsistency, imprecision, high dropout rates, and unclear risks of bias. Industry involvement can also be identified more specifically in key fluoxetine trials. The randomized trial by Michelson et al. [2] was supported by a grant from Lilly Research Laboratories, Eli Lilly and Company. The later randomized placebo-controlled trial by Michelson et al. [8] was also funded by Eli Lilly and Company; Michelson, Sarkar, and Pemberton were employees of the company, while the other listed investigators were paid consultants to Eli Lilly and Company. These relationships do not invalidate the findings, but they should be considered when interpreting the evidence and the need for independent replication.

Fourth, there is considerable uncertainty about long-term treatment. Bighelli et al. [1] in particular highlighted the need for longer-term follow-up and improved reporting of harms. Maintenance studies do exist, but the evidence is much less extensive than the acute-treatment literature.

Fifth, pharmacokinetic variability has yet to be successfully translated to individual clinical dosing. CYP2D6 influences the relationship between fluoxetine and norfluoxetine concentrations, but available evidence does not demonstrate a sufficiently consistent genotype-efficacy or genotype-tolerability relationship to inform routine prescribing.

Finally, the biggest evidence gap is related to advanced pharmaceutical formulations. Studies on cocrystals are still mainly physicochemical, while studies on nanoparticles and intranasal drug delivery are still mainly preclinical. There is no study available to show that patients with panic disorder have fewer panic attacks, fewer adverse effects, improved adherence, or better quality of life due to an advanced fluoxetine formulation.

Future Scope

A first step for future research should be to strengthen the clinical evidence base of fluoxetine in panic disorder. Contemporary randomized trials with current diagnostic criteria should examine panic frequency, panic-free status, anticipatory anxiety, agoraphobic avoidance, overall anxiety, functioning, quality of life, remission, adverse effects, and treatment discontinuation. This would be able to overcome one of the limitations identified by Bighelli et al. [1], who found limited long-term evidence and fairly little information regarding several patient-centered outcomes.

Dose optimization is a particularly promising area for further research. There is evidence that patients may differ in susceptibility to early adverse effects and in the dose needed for an adequate clinical response. Future adaptive trials could test whether titration based on tolerability and response leads to better treatment retention and remission than fixed-dose approaches.

Pharmacokinetic research should increasingly link genotype, drug exposure, and clinical outcome. Prospective studies in the future could genotype CYP2D6, repeatedly measure fluoxetine and norfluoxetine concentrations, record interacting medications and adherence, and determine whether these factors predict both efficacy and specific adverse effects. The next critical step is to see if using this information to guide treatment will improve patient outcomes.

Additional investigation may also be warranted for therapeutic drug monitoring. Since both fluoxetine and norfluoxetine contribute to pharmacological activity, interpretation of the parent-metabolite pattern may ultimately be more informative than measuring fluoxetine concentration alone. However, prospective validation is needed before routine clinical use of this approach.

Cocrystal research needs to go beyond showing altered dissolution. Future work should investigate whether physicochemical modifications lead to clinically meaningful changes in pharmacokinetics, dose requirements, tolerability, adherence, or exposure variability in humans.

Further translational steps are needed for nano-drug delivery and intranasal strategies. Preclinical studies should be continued to assess formulation stability, reproducibility, brain distribution, systemic exposure, nasal safety, and toxicity before transitioning to human pharmacokinetic studies and clinical trials.

A particularly attractive goal would be to determine whether targeted delivery can reduce systemic exposure while preserving or increasing therapeutically relevant central nervous system exposure. Such a result could provide a meaningful strategy for improving the benefit-risk profile of fluoxetine. But increased brain drug concentration alone would not be sufficient. Future studies need to demonstrate improvements in clinically meaningful outcomes.

Patient receptiveness should also be a formal treatment outcome. Further research should assess whether shared decision-making, information on early adverse effects, low-dose initiation, structured follow-up, flexible formulations, and individual titration are associated with a lower rate of premature discontinuation of treatment.

Ultimately, an effective personalized treatment approach may involve: right patient → appropriate medication → appropriate starting dose → appropriate rate of titration → acceptable adverse effect profile → sustained adherence → clinical response and remission.

Pharmacogenetics, therapeutic drug monitoring, or advanced delivery systems for drugs should be incorporated into this framework only when there is evidence that these improve one or more of these clinically meaningful stages. The relationship between current clinical optimization strategies, patient-specific factors, pharmacokinetic considerations, and emerging treatment approaches is summarized in Figure 1.

**Figure 1:**
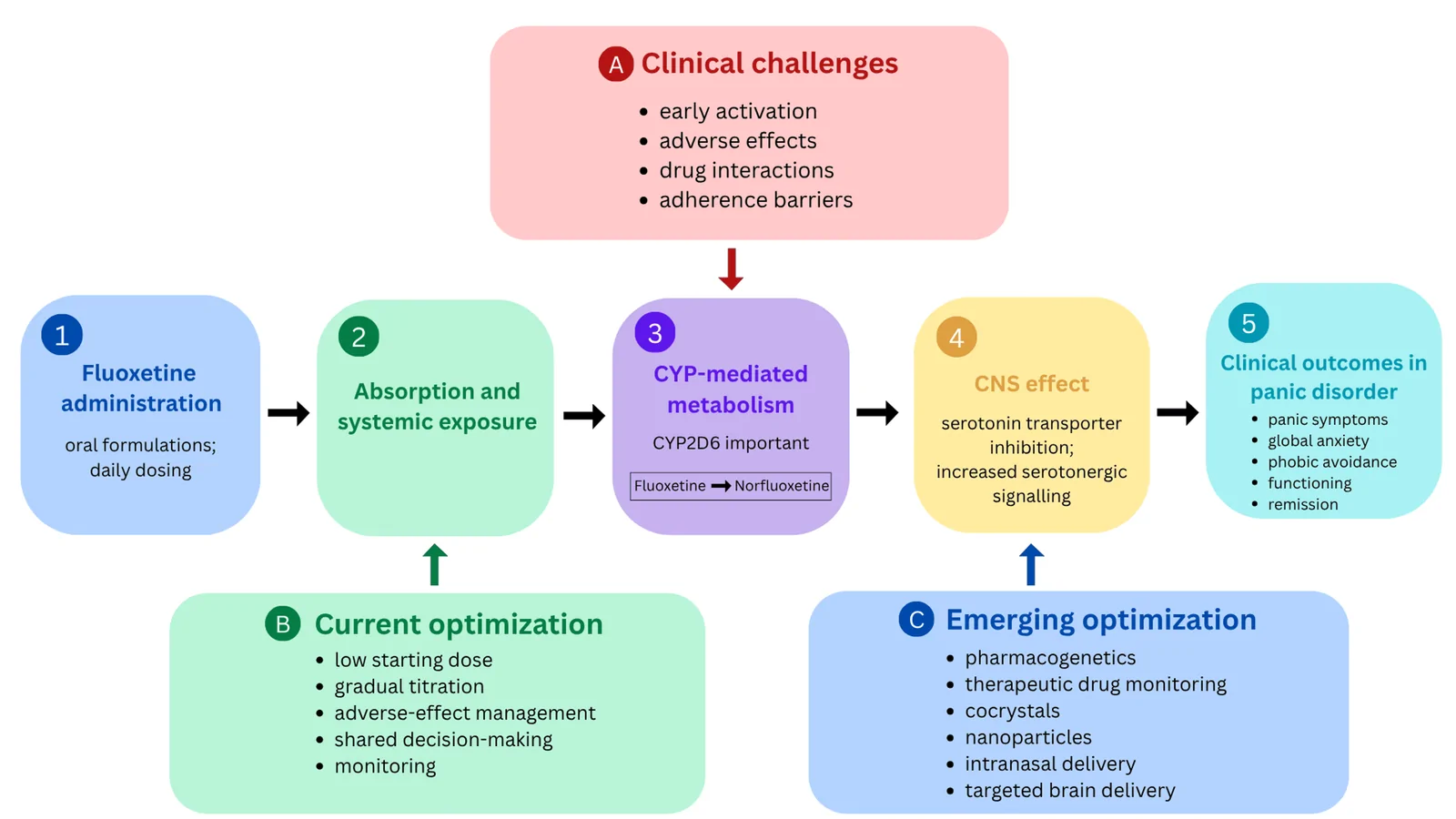
Conceptual pathway of fluoxetine treatment and optimization in panic disorder. CYP: cytochrome P450; CYP2D6: cytochrome P450 2D6; CNS: central nervous system. Image credits: Sehasbir Singh and Haseeb Mohammad

## Conclusions

Fluoxetine is an effective pharmacological option for the treatment of panic disorder, with benefits extending across panic symptoms and broader clinical outcomes such as anxiety, phobic symptoms, global improvement, and functioning. However, the available evidence does not consistently demonstrate that fluoxetine is superior to other SSRIs. Its clinical value therefore depends on balancing efficacy with tolerability and considering factors such as previous treatment response, comorbidities, drug interactions, adherence, and patient preference. Although fluoxetine is generally well tolerated when initiated and titrated appropriately, individual susceptibility to activation, gastrointestinal effects, insomnia, and other adverse effects may influence treatment continuation.

Pharmacokinetic variability provides a rationale for further individualization of fluoxetine treatment, but current evidence does not support routine genotype-guided dosing. Cocrystals, nanoparticles, intranasal drug delivery systems, pharmacogenetics, and therapeutic drug monitoring remain promising areas for future research rather than established clinical strategies. At present, treatment optimization should focus on individualized dosing, tolerability, adherence, patient preferences, and ongoing monitoring. Future research should determine whether pharmacogenetic, pharmacokinetic, and advanced drug delivery approaches can provide additional clinically meaningful benefits.
